# Proteomics reveals three molecular subtypes of Alzheimer's disease with distinct progression patterns

**DOI:** 10.1002/alz.71106

**Published:** 2026-02-06

**Authors:** Xiao‐He Hou, Wei Zhang, Kairan Kang, Yifei Jin, Peng Ren, Linbo Wang, Zeyu Li, Yuzhu Li, Jia You, Bei Zhang, Qing Ma, for the Alzheimer's Disease Neuroimaging Initiative, Fang Xie, Jin‐Tai Yu, Jian‐Feng Feng, Wei Cheng

**Affiliations:** ^1^ Department of Neurology Qingdao Municipal Hospital University of Health and Rehabilitation Sciences Qingdao China; ^2^ Institute of Science and Technology for Brain‐Inspired Intelligence, Department of Neurology, Huashan Hospital Fudan University Shanghai China; ^3^ State Key Laboratory of Brain Function and Disorders and MOE Frontiers Center for Brain Science Fudan University Shanghai China; ^4^ Key Laboratory of Computational Neuroscience and Brain‐Inspired Intelligence Fudan University, Ministry of Education Shanghai China; ^5^ Department of Nuclear Medicine & PET Center, Huashan Hospital Fudan University Shanghai China; ^6^ School of Data Science Fudan University Shanghai China; ^7^ Department of Computer Science University of Warwick Coventry UK; ^8^ Fudan ISTBI—ZJNU Algorithm Centre for Brain‐inspired Intelligence Zhejiang Normal University Zhejiang China

**Keywords:** Alzheimer's disease, biological subtypes, cerebrospinal fluid, proteomics

## Abstract

**INTRODUCTION:**

Alzheimer's disease (AD) shows marked molecular heterogeneity. Defining biological subtypes may refine diagnosis and treatment.

**METHODS:**

We analyzed cerebrospinal fluid (CSF) proteomics and longitudinal data from 550 participants in the Alzheimer's Disease Neuroimaging Initiative with up to 16.5 years of follow‐up. We profiled 6361 proteins, applied machine learning to identify biological subtypes, and validated them in three independent cohorts.

**RESULTS:**

Three AD subtypes were identified. Subtype 1, enriched in RNA metabolism pathways, showed the mildest atrophy and slowest cognitive decline. Subtype 2, characterized by axonogenesis‐related pathways, exhibited the greatest CSF tau elevations, moderate atrophy, and intermediate decline. Subtype 3, associated with catabolic processes, showed the most severe atrophy and fastest progression. These patterns were consistently replicated across validation cohorts.

**DISCUSSION:**

These findings demonstrate robust, biologically distinct AD subtypes linked to divergent molecular pathways, clinical features, and progression rates. Such refined stratification supports precision diagnostics and targeted therapeutic strategies.

## Highlights

- Large‐scale CSF proteomics and machine learning identified three robust biological subtypes of AD.
- Subtypes exhibit distinct molecular processes accompanied by divergent cognitive trajectories, brain atrophy patterns, and progression rates.
- Subtype 1 showed RNA metabolism dysregulation with relatively stable disease course; subtype 2 was enriched in axonogenesis pathways with intermediate progression; subtype 3 was driven by catabolic processes with rapid neurodegeneration.

## 1 BACKGROUND

Alzheimer's disease (AD) is a complex and progressive neurodegenerative disorder and the leading cause of dementia in the elderly, posing a significant public health challenge.^1^, ^2^ AD is primarily characterized by memory loss and cognitive decline and biologically defined by the deposition of amyloid beta (Aβ) and hyperphosphorylated tau tangles, but the pathogenesis of AD is much more complex beyond Aβ and tau.^3^, ^4^ Evidence has demonstrated that AD exhibits heterogeneity in various aspects, including amyloid composition, tau distribution, neuroimaging findings, and clinical symptoms.^4^ In addition, the progression trajectories of clinical and pathological features in AD patients differ across populations.^5^ However, the underlying molecular mechanisms contributing to the heterogeneity in AD remain poorly understood. Elucidating this heterogeneity to uncover the diverse pathophysiological pathways involved could facilitate targeted interventions and precision medicine approaches.

The clinical subtypes of AD have been investigated based on variations in cognitive profile, age of onset, and neuroimaging features.^6^, ^7^, ^8^, ^9^ Nevertheless, the underlying biological mechanisms of these subtypes remain unclear due to the lack of microscopic‐level information on molecular alterations. Previous studies primarily focusing on macro‐level phenotypes reported variable findings, which were likely influenced by multiple factors affecting clinical features. This variability highlights the need to investigate the biological subtypes of AD, as differences in clinical characteristics and progression trajectories may be driven by complex molecular processes that extend beyond the conventional paradigm.

Changes in cerebrospinal fluid (CSF) proteins directly reflect an individual's brain physiology and provide insight into the molecular mechanisms driving disease progression.^10^, ^11^ Protein profiling in CSF is particularly relevant in AD, as it captures physiological changes at early disease stages, even when cognition is still intact.^12^ CSF proteomics offers a broad perspective, encompassing proteins involved in diverse biological pathways. These approaches enable the identification of biological subtypes of AD with distinct molecular mechanisms, potentially revealing the underlying pathophysiological processes that contribute to differences in clinical features and disease progression. AD subtypes have been identified in studies utilizing CSF proteomics, which have predominantly focused on distinct molecular processes across subtypes.^13^, ^14^ These subtypes, however, exhibit only minor variation in terms of clinical presentation, cognitive decline, and neuroimaging findings, constraining their clinical utility. This limitation might result from the limited number of proteins used in these studies, restricting their abilities to capture subtle molecular differences among subtypes.

In this study, we employed a data‐driven approach to identify AD molecular subtypes using an extensive CSF proteomics dataset of 6361 proteins from the Alzheimer's Disease Neuroimaging Initiative (ADNI). The CSF protein profiles of the identified subtypes were interpreted through enrichment analyses in terms of biological processes. To investigate whether the CSF proteomic‐identified subtypes effectively reflected the clinical heterogeneity in AD, we compared the clinical and biological characteristics associated with AD, including cognitive function and decline, neuroimaging markers, core CSF biomarker profiles, and Polygenic Hazard Scores (PHSs), across subtypes to better define their distinct features. In addition, the clinical progression trajectories of subtypes assessed over a follow‐up period of up to 16.5 years were also compared. Finally, we replicated the results in three independent cohorts to confirm the robustness and reliability of our findings. Our study provides crucial insights into the biological underpinnings of AD, facilitating the development of precision interventions and personalized treatment strategies.

**RESEARCH IN CONTEXT**

1. **Systematic review**: Previous studies have applied cognitive, imaging, and CSF proteomics approaches to AD subtyping. CSF proteomics–based classifications have revealed different molecular processes across subtypes; however, these showed only minor differences in clinical presentation, cognitive decline, and neuroimaging findings, limiting their clinical applicability. A systematic definition of biologically distinct subtypes with consistent clinical and pathological correlates across cohorts has remained lacking.
2. **Interpretation**: Using large‐scale CSF proteomics integrated with longitudinal clinical and imaging data, we identified three robust and reproducible AD subtypes. Each subtype showed distinct molecular signatures, cognitive trajectories, and neuroanatomical patterns. These results extend previous classifications by linking proteomic heterogeneity to clinically meaningful differences, thereby refining our understanding of AD pathophysiology.
3. **Future directions**: Further research should validate these subtypes in more diverse, population‐based cohorts and examine their detectability in blood‐based biomarkers and multi‐omics datasets. Establishing subtype‐specific therapeutic responses will be essential to advance precision diagnostics and targeted interventions for AD.

## 2 METHODS

### 2.1 Study population

Data used in this study were downloaded from the ADNI database (http://adni.loni.usc.edu/) in August 2024.^15^ The ADNI project was launched in 2003 as a public‐private partnership and aims to examine whether serial imaging, genetic, other biological markers, and clinical and neuropsychological assessment can be combined to measure the progression of mild cognitive impairment (MCI) and early AD. The study was approved by the Institutional Review Boards (IRBs) of all participating centers, and written informed consent was obtained from all participants or their authorized representatives.

All the participants underwent standardized assessments and were classified as cognitively normal (CN), MCI, or dementia according to standard research criteria (www.adni‐info.org). The primary analysis of this study included 550 ADNI participants (150 CN, 271 MCI, and 129 dementia patients) with baseline CSF proteomic assay, CSF Aβ42, and CSF tau phosphorylated at threonine 181 (p‐tau181) data. AD was defined as the presence of abnormal amyloid markers (CSF Aβ42 < 1098 pg/ml, *n* = 465), and controls were defined as CN with normal amyloid and tau markers (CSF Aβ42 ≥ 1098 pg/ml and CSF p‐tau181 ≤ 26.64 pg/ml, *n* = 85).^16^, ^17^

### 2.2 CSF proteomics measurement

The CSF proteomics data used in the primary analysis of this study were acquired from the ADNI database. CSF samples were uniformly processed and stored at −80°C. The CSF proteomics levels of 7584 analytes were measured at baseline using the SOMAmer‐based capture array method (SomaScan 7K platform).^18^ The 7584 analytes were assayed using SomaLogic's SomaScan platform at once. All the data normalization steps were performed by SomaLogic, and the protein levels were reported as Relative Fluorescence Units (RFU). Hybridization normalization was performed at the sample level. Aptamers were then divided into three normalization groups based on the signal‐to‐noise ratio observed in technical replicates and samples, thereby avoiding combining features with different level of protein signal for additional normalization steps.^19^ Median normalization was then performed to eliminate other assay biases such as protein concentration, variation in reagent concentrations, and assay timing.^20^, ^21^ Finally, normalization to a reference was performed on individual samples using iterative adaptive normalization by maximum likelihood to account for additional technical and biological variation.

To ensure data quality, quality control (QC) was subsequently performed on the normalized SOMAscan7k data provided by SomaLogic using an in‐house protocol to detect and exclude outlier aptamers and samples.^19^, ^22^ Overall, at the end of QC, 7008 analytes that mapped to 6361 unique proteins were extracted and shared by the ADNI. Analytes with more than 10% missing rates were further excluded. Among multiple measurements for the same protein, we selected the SOMAmer that exhibited the largest difference between CN A−T− individuals and biologically defined AD groups. This strategy was adapted from a previously published approach for probe selection.^23^, ^24^ Finally, 6280 unique proteins were retained. Protein values were log_2_‐transformed and then scaled according to the mean and standard deviation of the control group to generate *z*‐scores. Positive and negative values indicate higher and lower than controls.

### 2.3 Magnetic resonance imaging

Preprocessed longitudinal structural MRI data were obtained from the ADNI database. Details of the imaging protocol and preprocess pipeline can be found in the documents at the ADNI depository (https://adni.loni.usc.edu/data‐samples/adni‐data/neuroimaging/mri/). Structural brain MRI data were acquired using T1‐weighted scans with volumetric magnetization‐prepared rapid gradient echo (MP‐RAGE) sequence. FreeSurfer was used for preprocessing.^25^ Gray matter volumes of 68 cortical brain regions were extracted based on the Desikan‐Killiany atlas,^26^ and the volumes of 14 subcortical brain regions were extracted based on the ASEG atlas.^25^

### 2.4 Cognitive assessments

Longitudinal cognitive assessments were extracted from the ADNI database. Global cognitive function and disease severity were assessed using the Mini‐Mental State Examination (MMSE) and Clinical Dementia Rating Scale Sum of Boxes (CDR‐SB) scores. The Alzheimer’s Disease Sequencing Project Phenotype Harmonization Consortium Phenotype Harmonization Consortium—Composite Cognitive Scores were used to assess specific cognitive domains, including memory, executive, language, and visuospatial function.^27^ Higher scores indicated better cognitive function.

### 2.5 Polygenic hazard score

The Desikan AD PHS was calculated based on a Cox proportional hazard regression model combining AD‐associated single nucleotide polymorphisms (SNPs).^28^ Specifically, it included 31 SNPs and two apolipoprotein E (*APOE*) variants (ε2/ε4). A higher PHS indicates a higher risk of AD. To evaluate polygenic risk independent of the *APOE* locus, we recalculated the PHS after removing all variants within the *APOE* gene region. Variants located on chromosome 19 between 45,384,477 and 45,432,606 (build 37/hg19) were excluded from the summary statistics.^29^ This region encompasses 10 kilobases upstream of the start site of TOMM40 (45,394,477) and 10 kilobases downstream of the end site of APOC1 (45,422,606), capturing the extended linkage disequilibrium block surrounding the *APOE* locus. The *APOE*‐excluded PHS was constructed based on genome‐wide association study results reported by Bellenguez et al.^30^

### 2.6 AD subtype discovery

Since this study focused on AD subtypes, AD‐related proteins were first selected. For each protein, the Kruskal–Wallis test was used to test the significant difference between the control and AD groups, and only proteins with *p* < 0.01 were retained (no correction for multiple comparisons). After this, 590 proteins were retained for subsequent subtype analysis. The value of each protein was further scaled to a non‐negative value between 0 and 1, and finally the NMF package in R was used to perform non‐negative matrix factorization analysis.^31^ The number of clusters was identified based on cophenetic correlation, residual sum of squares (RSS), and silhouette consensus.^31^ We analyzed clusters from two to 10. When the number of clusters is three, the cophenetic correlation is 0.87, which is higher than that of clusters from four to 10.^32^ When the number of clusters is three, the reduction of RSS compared to the lower cluster is the greatest, which equals 556.^33^ When the number of clusters is three, the silhouette consensus is 0.87, which is higher than that of clusters from four to 10.^34^ Based on these metrics and the biological interpretability of the resulting clusters, three was selected as the optimal solution.

### 2.7 Predicting AD subtypes in replication cohorts

Replication cohort 1 included participants with baseline CSF proteomic assay, CSF Aβ42, and CSF p‐tau181 data from the Parkinson's Progression Markers Initiative (PPMI) database (*n* = 232). All participants with Parkinson's disease (PD) were excluded. Population selection criteria have been described in detail elsewhere.^35^ AD was defined as the presence of abnormal amyloid markers (CSF Aβ42 < 683 pg/ml, *n* = 137), and controls were defined as CN with normal amyloid and tau markers (CSF Aβ42 ≥ 683 pg/ml and CSF p‐tau181 ≤ 24 pg/ml, *n* = 95).^36^ PPMI has enrolled participants across 33 clinical sites, including individuals recently diagnosed with PD, those at risk of developing PD (prodromal participants), and healthy controls. The PPMI study was approved by the IRBs of all participating centers, and written informed consent was obtained from all participants or their authorized representatives. Replication cohort 2 (*n* = 166) was described previously by Modeste et al.^37^ Participants were included from Emory's Goizueta Alzheimer's Disease Research Center (ADRC). AD was defined as the presence of abnormal amyloid markers (CSF Aβ42 < 1098 pg/ml, *n* = 125), and controls were defined as CN with normal amyloid and tau markers (CSF Aβ42 ≥ 1098 pg/ml and CSF p‐tau181 ≤ 19.2 pg/ml, *n* = 41).^16^ Replication cohort 3 included 149 AD and 125 controls from the Emory Goizueta ADRC and Emory Healthy Brain Study. AD was defined as the presence of abnormal amyloid markers (CSF Aβ42 < 338 pg/ml, *n* = 149), and controls were defined as CN with normal amyloid and tau markers (CSF Aβ42 ≥ 338 pg/ml and CSF p‐tau181 ≤ 90 pg/ml, *n* = 125). Details for replication cohort 1 were previously described by the PPMI study,^35^ replication cohort 2 by Modeste et al.,^37^ and replication cohort 3 by Dammer et al.^38^

To enable independent replication, a random forest classifier was trained in the discovery cohort using only proteins shared with each replication cohort with the randomForest package in R. Protein levels were *z*‐score‐normalized beforehand. The trained classifier was then directly applied to each replication cohort to predict subtype membership based on the highest probability. No retraining or model adjustment was performed in the replication cohorts (Figure S1). This approach ensured that the subtype definitions derived from the discovery cohort would be tested for generalizability in external datasets. Prior to prediction, protein levels in the replication cohorts were also z‐score‐normalized to account for cohort‐specific distributions.

### 2.8 Statistical analyses

To assess clinical progression, conversion to AD dementia was the endpoint event, defined as a change in diagnosis from CN or MCI to dementia based on ADNI clinical assessment protocol (https://adni.loni.usc.edu/help‐faqs/adni‐documentation/). Follow‐up years were calculated from baseline to the date of first diagnosis of AD dementia or the maximum follow‐up duration. Subtype differences in progression to dementia were examined using Cox proportional hazards models, adjusted for age, sex, years of education, and *APOE* ε4 status (carrier or non‐carrier). The *p* values were false discovery rate (FDR) corrected. The proportional hazard assumption was assessed using Schoenfeld residuals, and no violation was found.

To characterize the biological process of each subtype, linear models were first built for each protein to compare whether each subtype was significantly different from the control group. The subtype of the participants was used as a predictor, and the protein level was used as the outcome, corrected for age and sex. For each subtype, enrichment analysis was performed separately for proteins that were significantly higher than the control group and significantly lower than the control group (*p* < 0.05 after FDR correction). Proteins were mapped to genes according to the correspondence provided by ADNI, and Gene Ontology (GO) enrichment analysis, Kyoto Encyclopedia of Genes and Genomes (KEGG) pathway analysis, and WikiPathways analysis were performed using the clusterProfiler package in R,^39^, ^40^ with 6083 genes encoding the SomaScan 7k proteins as the background gene set. GO terms include Biological Process (BP), Molecular Function (MF), and Cellular Component (CC). The *p* values were obtained by Fisher's exact test and corrected for multiple testing with the FDR procedure.

For cognitive assessments, subtype differences at baseline were tested with linear models, and differences in longitudinal progression between subtypes were tested with linear mixed models. Age, sex, years of education, and *APOE* ε4 status were included as covariates, and *p* values were FDR corrected. Differences in CSF Aβ42, p‐tau181, and total tau (t‐tau) between subtypes were tested using linear models adjusted for age, sex, years of education, and *APOE* ε4 status, and *p* values were FDR corrected. For brain regional volumes, linear models were used to test differences between each subtype and the control group at baseline, and linear mixed models were used to test differences between each subtype and the control group during longitudinal progression. Age, sex, years of education, *APOE* ε4 status, and estimated total intracranial volume were included as covariates, and *p* values were FDR corrected.

## 3 RESULTS

### 3.1 Participant characteristics

**FIGURE 1:**
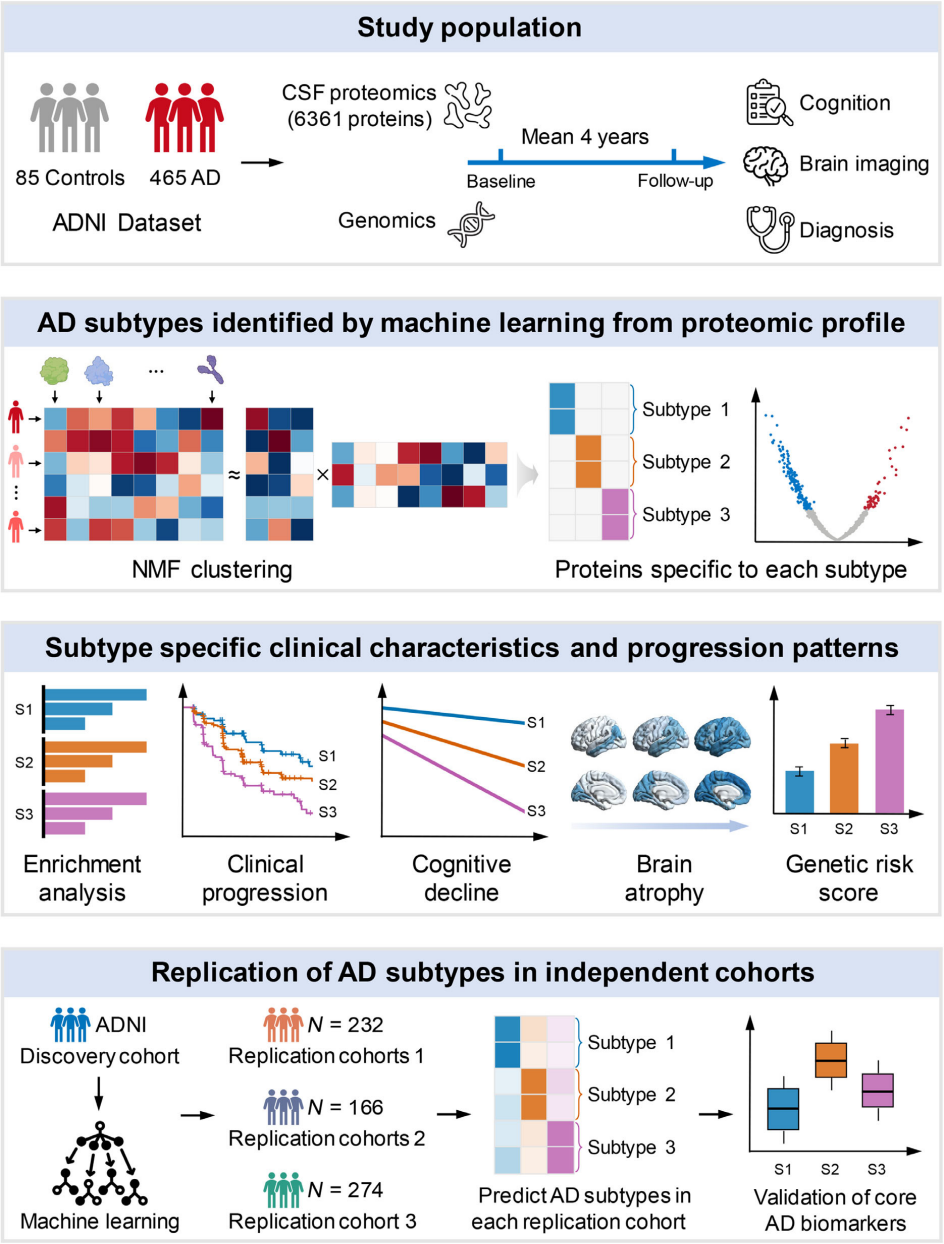
Graphical abstract. This study utilized cerebrospinal fluid (CSF) proteomic profiles (6361 proteins) and machine learning approaches to identify three distinct biological subtypes of Alzheimer's disease (AD) from the Alzheimer's Disease Neuroimaging Initiative dataset, comprising 465 AD patients and 85 controls with a mean follow‐up of 4 years. Subtypes were characterized by specific clinical and molecular features, including differences in genetic risk (*APOE* ε4 carrier rates and Polygenic Hazard Scores), cognition function, brain atrophy, and disease progression. Subtype‐specific molecular processes were identified through enrichment analysis, highlighting distinct pathophysiological mechanisms. The classification was validated in three independent cohorts (*N* = 232, *N* = 166, and *N* = 274), where subtype predictions were consistent with core CSF biomarkers and replication results further confirmed the robustness of the subtype classification.

The study included individuals from the ADNI cohort who had available CSF proteomics data. A total of 550 individuals were included in the study, comprising 85 control subjects and 465 individuals with AD across the clinical spectrum from the ADNI cohort. AD was defined by the presence of abnormal amyloid biomarkers (CSF Aβ42 < 1098 pg/mL), while controls were defined as CN individuals with normal amyloid and tau biomarkers (CSF Aβ42 ≥ 1098 pg/mL and CSF p‐tau181 ≤ 26.64 pg/mL).^16^, ^17^ Among the AD patients, there were 65 (13.98%) patients with normal cognition, 271 (58.28%) patients with MCI, and 129 (27.74%) patients with AD‐type dementia. AD patients were more likely to carry an *APOE* ε4 allele, had lower MMSE scores, and exhibited abnormal CSF p‐tau and t‐tau levels more frequently than control subjects. Age, gender, and educational level were similar between AD and control groups. CSF proteomics data included 6361 proteins, of which 590 proteins showed differential levels in AD patients compared to control subjects and were subsequently used for clustering analysis using non‐negative matrix factorization (NMF). The key elements of the study design and analytical framework are summarized in Figure 1.

### 3.2 Three biological subtypes identified by CSF proteomic profile

**FIGURE 2 alz71106-fig-0002:**
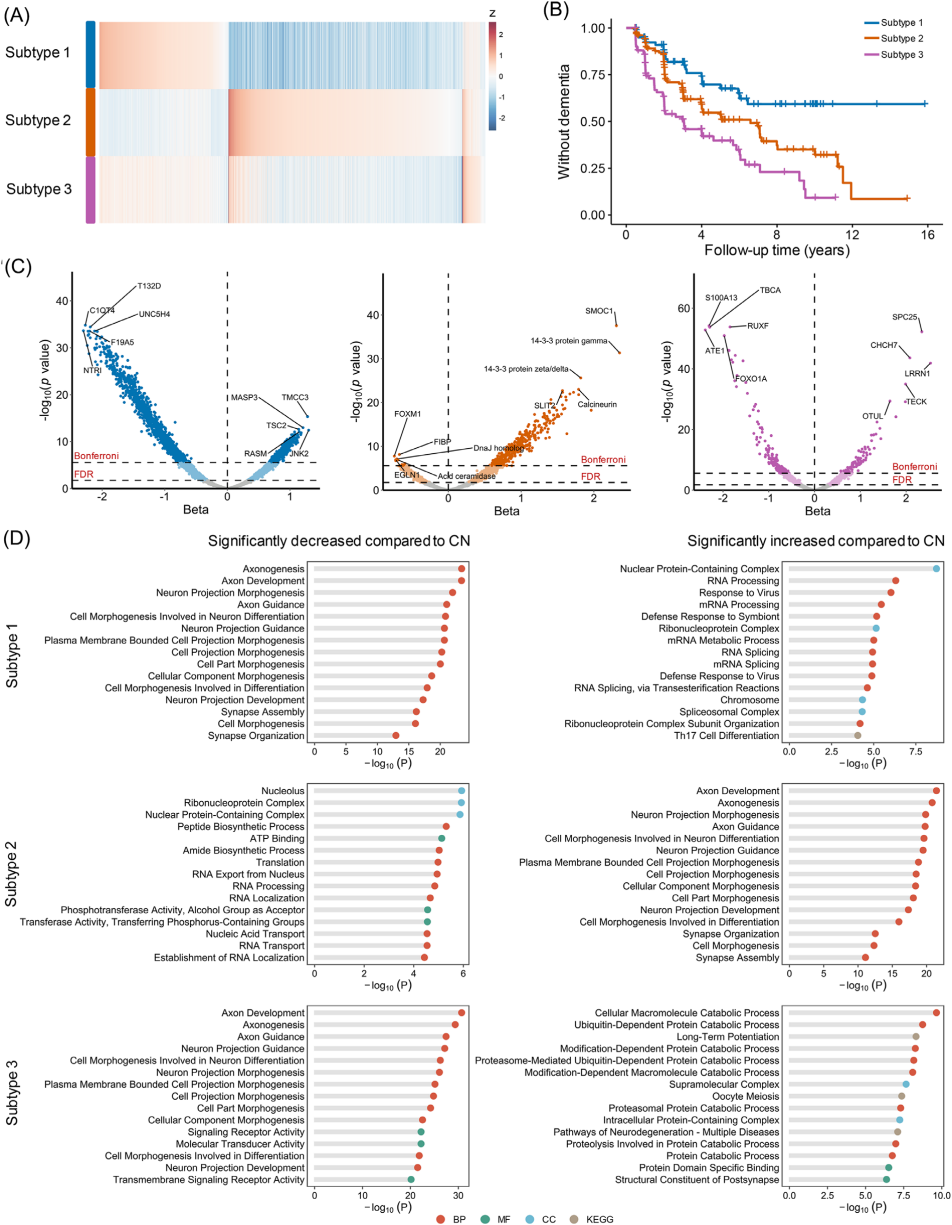
Three subtypes identified by CSF proteomic data and their biological pathways. (A) Cerebrospinal fluid protein levels (columns) averaged across individuals within subtypes (rows). (B) Different disease progression trajectories among the three subtypes. Subtype 1 exhibited the slowest progression to dementia, subtype 2 demonstrated an intermediate rate, while subtype 3 showed the most rapid progression. (C) Volcano plots showing β coefficient (*x*‐axis) and −log10(*p* value) (*y*‐axis) for proteins with significantly different levels between the three subtypes and the control group. (D) The enrichment analysis for the proteins of the three subtypes across Gene Ontology and Kyoto Encyclopedia of Genes and Genomes biological processes. Proteins increased in subtype 1 were enriched in RNA metabolism and processing pathways, while subtype 2 was characterized by proteins associated with axon growth and morphogenesis. In subtype 3, the enriched proteins were primarily involved in cellular catabolic processes.

To identify AD subtypes, proteins showing significant differences between AD and control groups (*p* < 0.01, Kruskal–Wallis test) were first selected. NMF was then applied to classify AD patients into distinct subtypes. The optimal number of clusters was determined to be three based on cophenetic correlation, RSS reduction, and silhouette consensus. When the number of clusters was set to three, the cophenetic correlation (0.87) and silhouette consensus (0.87) were higher than those for four to 10 clusters, and the reduction in RSS was the largest (556) compared to lower cluster numbers. Based on this approach, we identified three biological subtypes of AD patients based on their CSF proteomic profiles (Figure 2A). Subtype 1 included 97 participants with a mean age of 73.30 years, subtype 2 included 200 participants with a mean age of 73.74 years, and subtype 3 included 168 participants with a mean age of 74.32 years. Age, gender, and educational level did not show significant differences among the three subtypes (Table 1).

**TABLE 1:** Comparison of subtypes according to clinical characteristics.

Characteristic^a^	Controls *n* = 85	Subtype 1 *n* = 97	Subtype 2 *n* = 200	Subtype 3 *n* = 168	Statistics^b^
Age, years	73.59 (5.33)	73.30 (7.74)	73.74 (8.01)	74.32 (6.94)	F = 0.59 (*p* = 0.55)
Female, *n* (%)	39 (46)	41 (42)	89 (44)	62 (37)	χ^2^ = 2.22 (*p* = 0.33)
Education, years	16.22 (2.78)	16.09 (2.84)	15.98 (2.89)	15.78 (2.90)	F = 0.41 (*p* = 0.66)
Cognitive diagnoses (normal/MCI/dementia)	/	26/57/14	31/127/42	8/87/73	χ^2^ = 48.53 (*p* = 7.33×10^−^^10^)
*APOE* ε4 status (0/1/2)	73/12/0	78/18/1	86/99/15	3/96/69	χ^2^ = 208.04 (*p* = 7.02×10^−^^44^)
AD Polygenic Hazard Scores	−0.18 (0.50)	0.01 (0.63)	0.51 (0.75)	1.32 (0.60)	F = 119.15 (*p* = 9.48× 10^−^^42^)
CSF Aβ42, pg/ml	1661.89 (385.08)	712.20 (240.92)	736.11 (170.08)	577.43 (173.68)	F = 35.00 (*p* = 7.02× 10^−^^15^)
CSF p‐tau181, pg/ml	18.27 (4.01)	15.51 (5.45)	39.42 (15.74)	32.16 (12.79)	F = 105.68 (*p* = 1.83× 10^−^^38^)
CSF total tau, pg/ml	209.14 (45.14)	168.01 (50.75)	386.09 (137.48)	321.30 (118.16)	F = 111.25 (*p* = 4.04× 10^−^^40^)

Abbreviations: Aβ, amyloid beta; AD, Alzheimer's disease; CSF, cerebrospinal fluid; APOE, apolipoprotein E; MCI, mild cognitive impairment; p‐tau, tau phosphorylated at threonine 181;.

^a^ Continuous data are presented as mean (standard deviation) and categorical variables as number (percentage).

^b^ Differences across three AD subtypes were compared using analysis of variance (for continuous variables) and chi‐squared test (for discrete variables).

Subtype 1 exhibited increased levels of proteins including TMCC3, MASP3, TSC2, JNK2, and RASM and significantly decreased levels of NTRI, C1QT4, T132D, F19A5, and UNC5H4. Subtype 2 was characterized by elevated levels of SMOC1, 14‐3‐3 protein gamma, and 14‐3‐3 protein zeta/delta and decreased levels of FOCM1, FIBP, and EGLN1. Subtype 3 showed increased levels of SPC25, CHCH7, and LRRN1, with reductions in ATE1, S100A13, and TBCA (Figure 2C).

Enrichment analyses revealed distinct biological processes for each subtype (Tables S1–S6). Increased proteins in subtype 1 were enriched in the processes related to the nuclear protein‐containing complex, RNA processing, and mRNA processing (Figure 2D), indicating that subtype 1 was characterized by dysfunction in RNA metabolism and processing. The decreased proteins were predominantly involved in axonogenesis, axon development, and neuron projection morphogenesis. For subtype 2, proteins specifically increased in this subtype were enriched in axon development, axonogenesis, and neuron projection morphogenesis (Figure 2D), underscoring the disruption of axonal integrity. However, the downregulated proteins in this subtype were primarily associated with nuclear protein synthesis complex. Compared to controls, proteins increased in subtype 3 showed enrichment associated with cellular catabolism, including cellular macromolecule catabolic process, ubiquitin‐dependent protein catabolic process, long‐term potentiation, and modification‐dependent protein catabolic process. Similar to subtype 1, the decreased proteins were enriched for pathways related to axon development and axonogenesis.

We also performed pathway enrichment analysis using WikiPathways to further characterize the molecular features of the subtypes. For subtype 1, enriched pathways included growth factor receptor signaling and immune‐related processes, such as Ras signaling and B‐cell‐receptor signaling pathways. Subtype 2 was characterized by significant enrichment in axon guidance and synaptic function pathways. For subtype 3, enrichment was observed in pathways related to epidermal growth factor/epidermal growth factor receptor signaling, neurotrophic regulation, and the ubiquitin‐proteasome system.[Fig alz71106-fig-0002]

### 3.3 Cognitive function and progression trajectories in different AD subtypes

**FIGURE 3:**
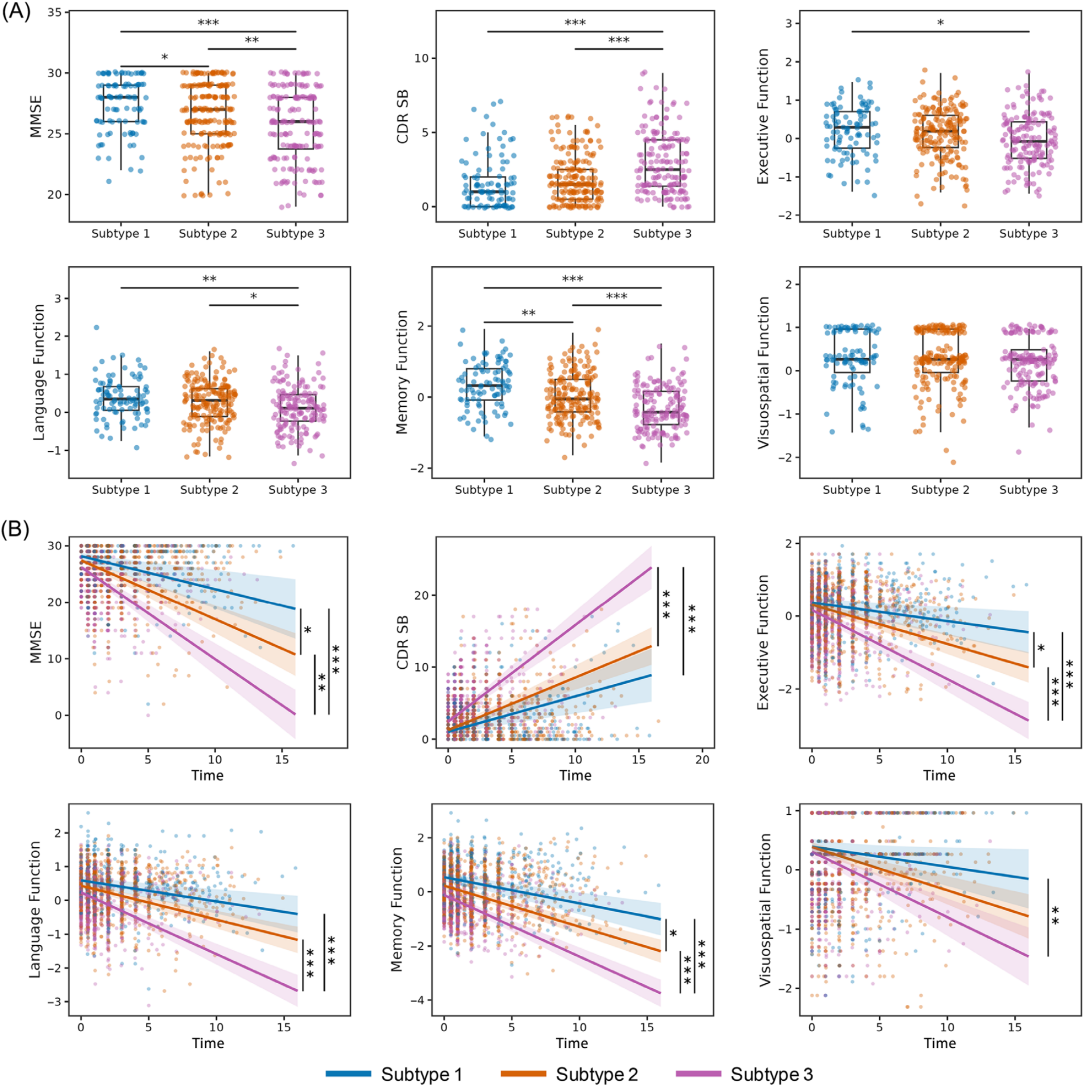
Cognition profiles of the three subtypes. (A) Baseline cognitive functions among the three subtypes. Significant differences were observed in MMSE, CDR‐SB, executive function, language function, and memory function at baseline among the subtypes, while visuospatial function did not show significant differences. (B) Cognitive decline during the follow‐up period differed significantly among the subtypes across multiple measures, including MMSE, CDR‐SB, executive function, language function, memory function, and visuospatial function. CDR‐SB, Clinical Dementia Rating Scale Sum of Boxes; MMSE, Mini‐Mental State Examination.

To investigate the differences in cognitive function and disease progression across the identified subtypes, we compared the neuropsychological test results and progression rates among subtypes (Figure 3). After adjusting for age, sex, years of education, and *APOE* ε4 status, subtype 3 had the highest CDR‐SB scores, indicating more severe dementia compared to subtype 1 (*p* = 3.47×10^−^^5^) and subtype 2 (*p* = 4.31×10^−^^6^) at baseline. We then compared the MMSE scores to investigate general cognition among the three subtypes. At baseline, subtype 1 had the highest MMSE score (*p* = 0.041 compared to subtype 2 and *p* = 7.95×10^−5^ compared to subtype 3), followed by subtype 2 (*p* = 0.002 compared to subtype 3), with subtype 3 having the lowest MMSE scores. Memory and executive scores followed a similar trend. Subtype 1 performed better than subtype 2 (*p* = 0.003) and subtype 3 (*p* = 8.24×10^−7^) in memory function, while subtype 2 also exhibited better memory function than subtype 3 (*p* = 2.54×10^−^^4^). For executive function, subtype 1 was also found to perform better than subtype 3 (*p* = 0.041). Language function scores were higher in subtype 1 (*p* = 0.003) and subtype 2 (*p* = 0.024) compared to subtype 3, while visuospatial function showed no difference among the subtypes (Figure 3A).

Subtype differences in progression to dementia were examined using Cox proportional hazards models, adjusted for age, sex, years of education, and *APOE* ε4 status. During the follow‐up period, clinical progression and cognitive decline rates varied differently across subtypes. As shown in Figure 2B, patients were all free from dementia at baseline and each subtype exhibited distinct trajectories of clinical progression. Subtype 3 patients progressed to dementia faster than subtype 1 (*p* = 0.022) and subtype 2 (*p* = 0.047). While the progression rate in subtype 2 was also faster than that in subtype 1, the difference was not statistically significant after adjustment (*p* = 0.117). Additionally, the rates of change in MMSE and CDR‐SB correspond to their respective clinical progression pattern (Figure 3B). Individuals in subtype 1 had the slowest MMSE decline rate (*p* = 0.027 compared to subtype 2; *p* = 3.10×^−6^ compared to subtype 3), while those in subtype 3 exhibited the fastest rates of decline (*p* = 0.02 compared to subtype 2). Similarly, the rate of CDR‐SB increase was most significant in subtype 3 (*p* = 8.42×10^−8^ compared to subtype 1; *p* = 2.16×10^−6^ compared to subtype 2). The decline trajectories of executive function, language function, memory function, and visuospatial function followed similar patterns during the follow‐up period (Figure 3B).

### 3.4 Different genetic risk profiles of AD subtypes

**FIGURE 4:**
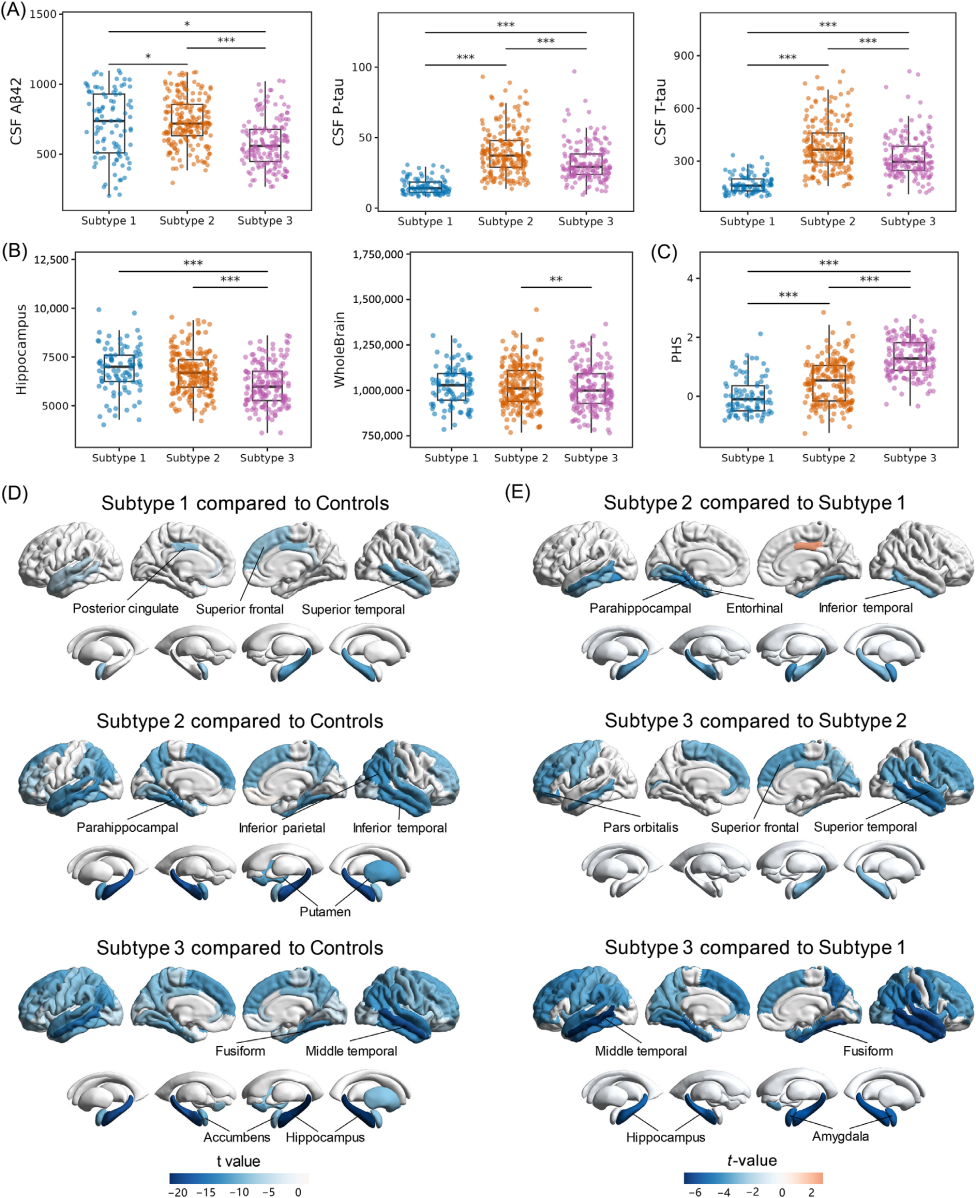
CSF biomarkers and brain atrophy features of the three subtypes. (A) Levels of CSF Aβ42, p‐tau, and t‐tau among the three subtypes. CSF Aβ42 levels were lower in subtype 3 than in subtype 1 and subtype 2. P‐tau and t‐tau levels were highest in subtype 2, with subtype 3 showing intermediate levels. (B) Subtype comparisons of the baseline volume of the hippocampus and whole brain. Subtype 3 exhibited lower baseline hippocampal volume compared to subtype 1 and subtype 2, with differences in whole brain volume also observed among the subtypes. (C) Differences in PHS among the three subtypes. Subtype 1 exhibited the lowest PHS, followed by subtype 2 with intermediate levels, and subtype 3 showed the highest PHS. (D) Comparison of longitudinal brain atrophy rates between the three subtypes and the control group. Compared to the control group, the three subtypes exhibited different atrophy rates in brain regions, with subtype 3 showing the most severe longitudinal brain atrophy and subtype 1 showing the mildest. (E) Comparison of longitudinal brain atrophy rates among the three subtypes. Among the three subtypes, subtype 3 exhibited the fastest brain atrophy rates, followed by subtype 2, while subtype 1 showed the slowest progression. Aβ, amyloid beta; CSF, cerebrospinal fluid; PHS, Polygenic Hazard Score; p‐tau, phosphorylated tau; t‐tau, total tau.

We first examined the differences in *APOE* ε4 carrier rates among the three groups. Subtype 1 exhibited the lowest *APOE* ε4 carrier rate at 19.59%, followed by subtype 2 at 57%, and subtype 3 with the highest carrier rate at 98.21% (Table 1). Consistently, the proportion of *APOE* ε4 homozygotes was highest in subtype 3 (43.5%), followed by subtype 2 (7.5%), and lowest in subtype 1 (1.0%). To further investigate the differences in genetic risk for AD, we compared PHS, as shown in Figure 4C. Subtype 1 had the lowest PHSs among the subtypes (*p* = 2.15×10^−^^8^ compared to subtype 2; *p* = 3.40×10^−^^4^ ^0^ compared to subtype 3), whereas subtype 3 had the highest PHSs (*p* = 1.12×10^−^^2^ ^6^ compared to subtype 2). After adjusting for *APOE* ε4, the PHS scores in subtype 3 remained significantly higher than those in subtype 1 (*p* = 4.25×10^−5^) and subtype 2 (*p* = 2.65×10^−7^).

Given the differences in *APOE* ε4 carrier frequency across the three subtypes, we recalculated a PHS after excluding variants within the *APOE* region to evaluate whether subtype‐specific differences in genetic risk extended beyond *APOE* ε4. The overall pattern of PHS differences remained consistent with the original analysis: subtype 3 exhibited the highest PHS, followed by subtype 2 and then subtype 1. Subtype 3 showed significantly higher PHS compared to both subtype 1 (*p* = 0.001) and subtype 2 (*p* = 0.002). Although subtype 2 had a higher PHS than subtype 1, this difference did not reach statistical significance (*p* = 0.302). These results suggest that the differences in genetic risk across subtypes are not solely attributable to *APOE* ε4 status.

### 3.5 Comparison of AD pathology patterns among subtypes

To investigate differences in AD pathology among the three subtypes, we compared the core CSF biomarkers of AD. The levels of CSF Aβ42, t‐tau, and p‐tau varied significantly across the groups (Figure 4A). Subtype 3 demonstrated lower levels of CSF Aβ42 than both subtype 1 (*p* = 0.011) and subtype 2 (*p* = 1.45×10^−8^). And individuals in subtype 3 had higher levels of CSF t‐tau (*p* = 1.13×10^−10^) and p‐tau (*p* = 1.29×10^−9^) than individuals in subtype 1. However, it is noteworthy that subtype 2 had the highest levels of CSF t‐tau (*p* = 3.32×10^−33^ compared to subtype 1; *p* = 3.43×10^−8^ compared to subtype 3) and p‐tau (*p* = 1.38×10^−31^ compared to subtype 1; *p* = 2.50×10^−8^, compared to subtype 3), though it is not the most severe subtype regarding the cognitive function and disease progression.

### 3.6 Comparison of brain atrophy pattern across the three AD subtypes

Differences in cortical and subcortical volumes among the three subtypes were investigated. Compared to controls, subtype 1 showed significant atrophy in 20 brain regions, subtype 2 in 19 brain regions, and subtype 3 in 21 brain regions at baseline (Table S7). All three subtypes showed significant brain atrophy, including the right amygdala, bilateral hippocampus, bilateral entorhinal cortex, and bilateral inferior temporal lobe, compared to controls. In addition, the individuals in subtypes 2 and 3 had significant atrophy in the left amygdala and the bilateral middle temporal lobe (Table S7). The brain atrophy profiles among the three subtypes were also compared. Subtype 3 exhibited the most severe brain atrophy at baseline, particularly in the hippocampus (*p* = 1.29 × 10^−^^4^ compared to subtype 1, *p* = 1.58 × 10^−^^5^ compared to subtype 2; Figure 4B), entorhinal cortex (*p* = 4.44 × 10^−^^4^ compared to subtype 1, *p* = 3.97 × 10^−^^5^ compared to subtype 2), fusiform gyrus (*p* = 0.045 compared to subtype 1, *p* = 0.001 compared to subtype 2), and middle temporal lobe (*p* = 0.026 compared to subtype 1, *p* = 0.039 compared to subtype 2). While subtype 2 exhibited more severe atrophy compared to subtype 1, the differences were not statistically significant.

Brain atrophy progression during the follow‐up also differed. Subtype 1 showed significantly faster atrophy rates in 12 regions, including the right hippocampus and the bilateral middle temporal lobe, compared to controls. In subtype 2, a total of 39 brain regions exhibited faster atrophy rates, with the most significant changes in the bilateral hippocampus, the left parahippocampal gyrus, and the left middle temporal lobe. Subtype 3 demonstrated the most severe brain atrophy rates, with 61 brain regions showing faster atrophy than in controls (Figure 4D and Table S8). The brain atrophy progression during the follow‐up period among the three subtypes was similar to the profiles at baseline. Subtype 3 showed the most severe atrophy progression compared to subtypes 1 and 2 across many brain regions (Figure 4E and Table S9). Subtype 2 also exhibited faster atrophy rate in 12 brain regions than subtype 1, including the bilateral hippocampus, bilateral fusiform, and left entorhinal (Figure 4E and Table S9).

### 3.7 Sensitivity analyses of AD subtypes

To demonstrate that the identified subtypes represent distinct biological entities rather than reflecting different stages of a single disease continuum or being driven by APOE ε4 carrier differences, we conducted complementary analyses.

Given the differences in *APOE* ε4 carrier frequency across subtypes, we performed an *APOE* ε4‐stratified analysis to assess whether subtype‐specific patterns persisted within *APOE* ε4‐homogeneous groups. In *APOE* ε4 carriers, pathway enrichment results were consistent with those in the full cohort. Subtype 1 remained enriched for RNA metabolism‐related pathways, subtype 2 for axonogenesis and neuronal projection development, and subtype 3 for catabolic and structural remodeling processes (Table S10). In *APOE* ε4 non‐carriers, subtype 1 maintained the original enrichment patterns. For subtype 2, several associated pathways, including vesicle‐mediated transport, synaptic signaling, and protein catabolic processes, were significantly enriched, supporting biological processes essential for axon growth and synaptic connectivity. Subtype 3 also largely preserved its initial profile, with significant enrichment in cellular catabolic processes, such as the proteasome accessory complex and endopeptidase complex, being consistently observed (Table S10).

Importantly, in both *APOE* carriers and non‐carriers, the CSF biomarker profiles showed similar patterns of subtype differences, broadly resembling those observed in the full cohort (Figure S2). In *APOE* ε4 non‐carriers, significant differences in CSF tau biomarkers were observed across the three subtypes, with subtype 2 exhibiting markedly elevated levels of both p‐tau and t‐tau compared to subtypes 1 and 3, suggesting more pronounced tau pathology (Figure S2A). CSF p‐tau levels were significantly higher in subtype 2 than in subtype 1 (*p* = 1.13 × 10^−12^) and subtype 3 (*p* = 3.22 × 10^−11^); similarly, t‐tau levels were significantly higher in subtype 2 than in subtype 1 (*p* = 3.05 × 10^−13^) and subtype 3 (*p* = 3.22 × 10^−11^). A similar pattern was found in *APOE* ε4 carriers, where subtype 2 again showed the highest levels of both p‐tau and t‐tau (Figure S2B). Specifically, p‐tau levels were significantly higher in subtype 2 compared to subtype 1 (*p* = 8.81× 10^−18^) and subtype 3 (*p* = 1.17× 10^−6^); t‐tau levels were also significantly higher in subtype 2 compared to subtype 1 (*p* = 6.47× 10^−19^) and subtype 3 (*p* = 1.46× 10^−6^).

To further assess whether the identified subtypes represent distinct biological entities rather than different stages of AD progression, we conducted two complementary analyses. First, we compared cognitive performance, brain imaging measures, PHS, and CSF tau levels across subtypes after adjusting for baseline CSF Aβ concentrations as a covariate in the entire sample. Baseline CSF Aβ was adjusted for as an indirect indicator of disease progression, given that amyloid accumulation typically precedes other pathological events. After accounting for baseline amyloid burden, the results remained consistent with the original findings. Subtype 1 showed better cognitive performance, slower brain atrophy progression, and lower CSF tau levels. Subtype 2 presented moderate cognitive and structural changes but higher tau levels. Subtype 3 exhibited poorer cognitive performance, faster brain atrophy progression, and intermediate tau levels (Figures S3 and S4; Tables S11–S15).

Second, we conducted a separate clustering analysis restricted to individuals with MCI at baseline. The clustering procedure was reapplied within this MCI‐only subgroup. Comparison with the original subtyping revealed consistent molecular features across stages. Subtype 1 consistently showed enrichment for RNA metabolism and processing pathways. Subtype 2, at the MCI stage, remained predominantly enriched for protein degradation pathways, particularly those related to the ubiquitin–proteasome system, which is critical for maintaining axonal integrity. Subtype 3 demonstrated strong enrichment for protein catabolic processes (Table S16). In the MCI subgroup, significant differences in CSF tau biomarkers were observed across the three subtypes. Subtype 2 exhibited substantially higher levels of both p‐tau and t‐tau compared to subtype 1 (*p* = 9.27 × 10^−12^ for p‐tau; *p* = 2.54 × 10^−13^ for t‐tau). Although p‐tau and t‐tau levels in subtype 2 were also higher than those in subtype 3, these differences did not reach statistical significance (*p* = 0.680 for p‐tau; *p* = 0.680 for t‐tau). These findings further support the idea that the identified subtypes reflect distinct biological processes rather than different stages of disease severity (Figure S5).

### 3.8 Replication of AD subtypes in independent cohorts

**FIGURE 5:**
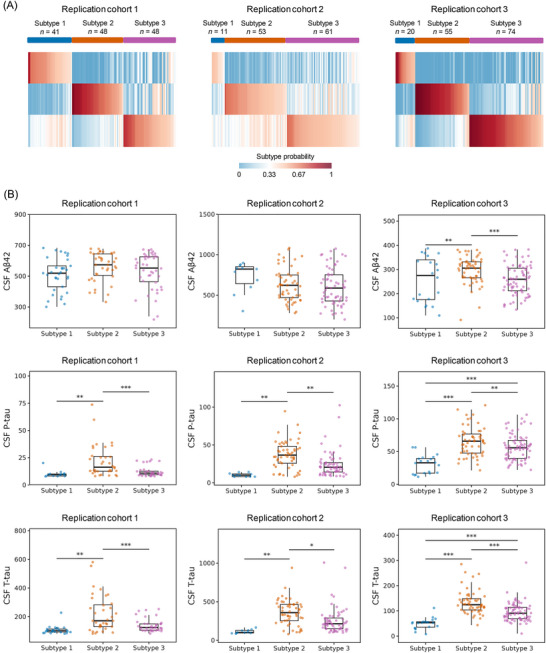
Validation in replication cohorts. (A) Subtype probability for each individual in three replication cohorts. All three subtypes were observed in the three replication cohorts with high subtype‐specific probabilities. (B) CSF Aβ42, p‐tau, and t‐tau among the three subtypes in the replication cohorts. Subtype 2 exhibited significantly higher levels of p‐tau and t‐tau compared to subtype 1 and subtype 3, consistent with findings from the discovery cohort. Aβ, amyloid beta; CSF, cerebrospinal fluid; p‐tau, phosphorylated tau; t‐tau, total tau.

To evaluate the reproducibility of the identified subtypes, we applied random forest classifiers trained in the discovery cohort (ADNI) to three independent replication cohorts.^35^, ^37^, ^38^ All replication cohorts were clinic‐based studies, with participants recruited from specialized research centers. Only proteins shared between the discovery and replication datasets were used for prediction. The replication cohort used only participants with AD for subtyping analysis (defined as the presence of abnormal amyloid biomarkers). Each individual in the replication cohorts was assigned to the subtype with the highest predicted probability. Using the random forest classifiers trained on the discovery data, we identified the three subtypes in three independent replication cohorts with available CSF proteomic data (Figure 5A). Across the replication cohorts, the subtype distributions were as follows: in replication cohort 1, 41 individuals (29.9%) were classified as subtype 1, 48 (35.0%) as subtype 2, and 48 (35.0%) as subtype 3; in replication cohort 2, 11 individuals (8.8%) as subtype 1, 53 (42.4%) as subtype 2, and 61 (48.8%) as subtype 3; in replication cohort 3, 20 individuals (13.4%) as subtype 1, 55 (36.9%) as subtype 2, and 74 (49.7%) as subtype 3. In the replication cohorts, demographic factors and clinical status were compared across the identified subtypes (Tables S17–S19). No significant differences in age or sex distribution were observed among the subtypes in most cohorts. Similar to the discovery cohort, *APOE* ε4 burden varied across subtypes in the replication cohorts, with subtype 3 showing the highest proportion of *APOE* ε4 carriers and subtype 1 the lowest. The overall trend in *APOE* ε4 carrier rates was consistent with that observed in the discovery sample.

Comparisons of CSF t‐tau and p‐tau levels across subtypes in the replication cohorts revealed patterns largely consistent with the main analyses (Figure 5B). In replication cohort 1, the levels of p‐tau and t‐tau in subtype 2 were higher than those in subtype 1 (*p* = 0.01 for p‐tau; *p* = 2.00 × 10^−3^ for t‐tau) and subtype 3 (*p* = 7.72 × 10^−6^ for p‐tau; *p* = 5.61 × 10^−7^ for t‐tau). Similarly, in replication cohort 2, subtype 2 exhibited elevated levels of p‐tau and t‐tau compared to subtype 1 (*p* = 1.28 × 10^−3^ for p‐tau; *p* = 1.28 × 10^−3^ for t‐tau) and subtype 3 (*p* = 1.28 × 10^−3^ for p‐tau; *p* = 0.01 for t‐tau). In replication cohort 3, the levels of p‐tau and t‐tau in subtype 2 are also higher than those in subtype 1 (*p* = 3.68 × 10^−8^ for p‐tau; *p* = 1.29 × 10^−11^ for t‐tau) and subtype 3 (*p* = 8.54 × 10^−3^ for p‐tau; *p* = 2.17 × 10^−7^ for t‐tau). These results confirmed that the subtypes identified in the discovery cohort were also observed in the replication cohorts.

**FIGURE 6:**
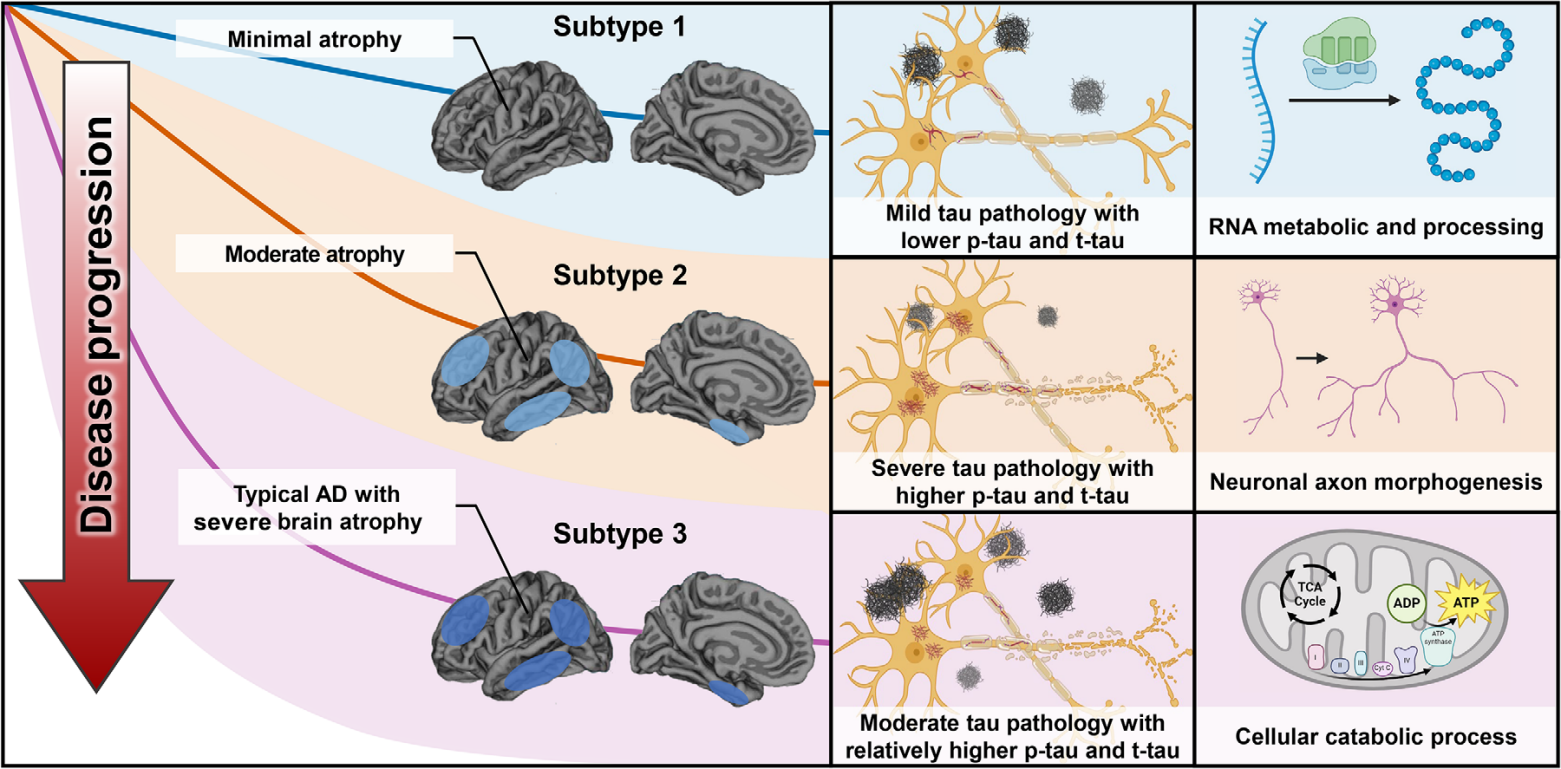
Summary of characteristics of the three Alzheimer's disease AD biological subtypes. Subtype 1 is associated with mild brain atrophy, slower cognitive decline, and dysregulation in RNA metabolism and processing. Subtype 2 is characterized by severe tau pathology, moderate brain atrophy rates, and axonogenesis‐related features. Subtype 3 exhibits the most severe brain atrophy, rapid disease progression, and enrichment in cellular catabolic processes.

In summary, our analyses identified three biologically and clinically distinct AD subtypes based on CSF proteomic profiles. These subtypes exhibited consistent differences across multiple modalities, including cognitive performance, rates of clinical progression, cortical atrophy patterns, genetic risk profiles, and core AD biomarkers (Figure 6).

## 4 DISCUSSION

In this study, we identified three distinct biological subtypes of AD based on large‐scale proteomics analysis, which were consistently validated across three large independent cohorts. Importantly, these subtypes exhibited significant differences in clinical progression, cognitive profiles, brain atrophy pattern, and core CSF biomarkers profile. Enrichment analysis further revealed that each subtype was characterized by proteins associated with specific biological pathways. In addition, unique genetic risk profiles of each subtype supported that the CSF‐based subtypes reflect distinct molecular mechanisms driving AD pathogenesis.

The subtypes identified in our study, defined by distinct CSF protein profiles, are associated with diverse biological processes. In subtype 1, proteins that were increased are enriched in RNA metabolism and processing pathways. Dysregulation of RNA and RNA‐binding proteins has been implicated in AD.^41^, ^42^ Notably, previous research suggested that specific circular RNAs were crucial in regulating Aβ production, metabolism, autophagy, and neuroinflammatory pathways.^43^ Furthermore, changes in mRNA translation changes in AD have been suggested to contribute to the pathological changes in AD, including Aβ and tau pathologies.^44^ Our findings that elevated proteins in subtype 1 are primarily involved in RNA processing, RNA splicing, and mRNA metabolism underscores the potential impact of RNA dysregulation in this subtype. In contrast, subtype 2 is characterized by proteins enriched in axon growth and morphogenesis, including axon development, axonogenesis, and neuron projection morphogenesis. Substantial evidence suggests that defects in axonal transport play a significant role in the pathogenesis of AD.^45^ Moreover, axonal dysfunction is closely associated with tau pathology, as abnormal tau accumulates within axons, forming neurofibrillary tangles that disrupt axonal transport.^46^ For subtype 3, the proteins were enriched in cellular catabolic process pathways. Autophagy is a crucial cellular degradation process that removes damaged organelles and protein aggregates through lysosomal activity.^47^ Clinically, individuals in subtype 3 exhibit the most severe cognitive decline and disease progression. Dysfunction in autophagy and lysosomal pathways might contribute to accelerated neurodegeneration, with previous studies reporting that autophagy/lysosomal dysfunction is involved in the pathogenesis of AD.^48^ In contrast to the enriched pathways associated with increased proteins, we also observed distinct biological pathways among proteins that were decreased in each subtype. In subtypes 1 and 3, decreased proteins were significantly enriched in pathways related to axonogenesis and axon development, which are also upregulated in subtype 2. This reciprocal pattern suggests that subtypes 1 and 3 may be characterized by reduced engagement of axon‐related pathways, whereas subtype 2 may show increased activity in axonal remodeling processes. In subtype 2, the decreased proteins were instead enriched in nuclear protein synthesis complex pathways, suggesting suppression of nuclear translational machinery in this subtype.

Previous studies of AD subtypes using CSF proteomics focused primarily on pathophysiological aspects with limited attention to differences in clinical features,^13^, ^14^ leaving the underlying biological mechanisms driving such heterogeneity largely underexplored. In contrast, our study leveraged a broader proteomic profile, enabling more comprehensive capture of biological diversity across individuals with AD. This broader proteomic coverage allowed us to identify three biologically distinct subtypes that differ in molecular pathways and exhibit consistent differences in clinical characteristics, biomarker profiles, and disease progression patterns. Specifically, individuals in subtype 1 (RNA dysregulation subtype) exhibited the mildest cognitive impairment and have the slowest rate of MMSE decline and progression to AD dementia. Individuals in subtype 3 (cellular catabolism subtype), on the other hand, were associated with the most severe cognitive deficits and rapid disease progression, while subtype 2 (axonogenesis‐related subtype) exhibited an intermediate profile with moderate cognitive impairment and clinical progression rate. Although our subtype 1 shares similarities with subtype 3 in the previous study by Tijms et al.,^14^ as both are related to RNA‐related processes, there are notable differences between the two studies. In our analysis, subtype 1 exhibited the mildest levels of Aβ and tau pathology, whereas their subtype 3 was associated with the highest tau burden. Furthermore, our tau‐dominant subtype (subtype 2) was characterized by proteins related to axonogenesis, suggesting that elevated tau levels in this group may reflect axonal injury or maladaptive remodeling processes. In addition, the consistent clinical differences across our subtypes underscore the greater heterogeneity of AD captured in our study and may provide important insights for future efforts in stratified prognosis and subtype‐specific therapeutic strategies.

Although the three identified AD subtypes exhibited differences in disease severity, clinical progression, and biomarker levels, several complementary analyses confirmed that these subtypes did not simply represent different stages along a single disease continuum. Adjusting for baseline CSF Aβ concentrations, which reflect early amyloid pathology and serve as a proxy for disease progression, did not alter the main findings. Moreover, when the clustering procedure was repeated among participants with MCI at baseline, the same molecular patterns were preserved. This consistency indicates that the observed subtype differences are not attributable to the degree of amyloid accumulation or overall disease severity. In addition, subtype 2 exhibited higher tau levels than both subtypes 1 and 3, yet its clinical and structural changes were only moderate, further suggesting that the three subtypes represent distinct biological entities rather than different disease stages. APOE ε4‐stratified analyses also demonstrated that the identified biological heterogeneity could not be explained solely by APOE genotype distribution.

Although the traditional definition of AD retains an amyloid‐centric approach,^3^ where amyloid pathology occurs first, followed by subsequent pathological changes in tau, a tau‐first subtype of AD was reported in previous studies.^49^, ^50^ This subtype is characterized by tau pathology with relatively mild amyloid deposition. However, the mechanisms underlying this unique subtype remain unclear. In our study, brain atrophy patterns and CSF biomarkers generally aligned with the clinical features of the three subtypes, except for subtype 2 (axonogenesis‐related subtype). Subtype 2 exhibited the highest levels of CSF p‐tau and t‐tau but showed less severe cognitive deficits, slower clinical progression, and milder brain atrophy. This observation contrasts with previous findings that link tau pathology closely to AD progression and cognitive impairment.^51^, ^52^ Given the strong association between axonal dysfunction and tau pathology, one possible explanation for this discrepancy is that elevated tau levels in subtype 2 may reflect axonal integrity disruption rather than direct neurodegeneration. These results suggest that while tau pathology plays a significant role in AD, other biological mechanisms, such as axonal dysfunction, also contribute to disease development. Thus, understanding the interaction between tau pathology and axonal dysfunction is important for uncovering the pathogenesis of this subtype and providing valuable insights into the mechanisms underlying tau‐first AD. This combination of factors could represent a critical pathway driving disease progression in these cases.

Our findings demonstrated significant differences in genetic risk profiles among the three subtypes. Subtype 1 exhibited the lowest PHS and *APOE* ε4 carrier rates, while subtype 3 showed the highest, with subtype 2 demonstrating intermediate PHS and *APOE* ε4 carrier rates between these two groups. Consistent with previous studies showing correlations between AD polygenic risk scores and markers of AD neurodegeneration as well as clinical progression,^28^, ^53^ our study revealed that higher PHS was associated with increased amyloid burden, faster brain atrophy rate, and disease progression across subtypes, further reinforcing that each CSF AD subtype corresponded to specific underlying molecular mechanisms. The distinct patterns of molecular processes and AD genetic risk profiles suggest that each AD subtype may benefit from tailored treatment approaches.

To strengthen the robustness of our findings, we validated them in three independent cohorts. All the subtypes were identified in the replication cohorts, and CSF biomarker profiles were consistent with those observed in the discovery cohort. These replications confirmed that the pathology of tau was strongly associated with the subtypes identified in our study. This association emphasizes the role of these pathological processes in the heterogeneity observed in AD. While the core biological features of the subtypes are reproducible, their relative frequencies may vary across populations. The observed differences in subtype proportions among the replication cohorts are likely attributable to differences in cohort design, population characteristics, and inclusion criteria. Despite these variations in subtype proportions, the core molecular and clinical features of each subtype remained consistent across cohorts, supporting the robustness of the subtype definitions. This validation reinforces the reliability of our subtype classification and underscores the utility of proteomics in distinguishing AD subtypes.

Several potential limitations should be taken into account when interpreting our results. First, while the ADNI dataset provides a comprehensive assessment of circulating proteins, it does not capture the entirety of the human proteome. Additionally, potential selection bias may influence the prioritization of secreted proteins for measurement. Second, the absence of longitudinal data in the validation cohorts prevents us from evaluating disease progression across the identified subtypes. Although cross‐sectional data allowed the identification of subtype‐specific patterns and their validation across independent cohorts, the lack of temporal information limits our ability to confirm whether these subtypes exhibit distinct progression trajectories. Future studies incorporating longitudinal data is essential to address this gap and enhance our understanding of the role of these subtypes play in AD progression.

In conclusion, we have identified three biological subtypes of AD using an extensive CSF proteomics analysis, revealing not only distinct molecular signatures but also corresponding differences in clinical features and disease progression. These findings highlight that the heterogeneity observed in AD patients is likely driven by variations in the underlying complex pathogenic mechanisms such as RNA metabolism and processing, neuronal axon morphogenesis, and cellular catabolic processes. Understanding the specific biological mechanisms driving each subtype is essential for the development of targeted therapeutic interventions, advancing precision medicine in AD. By offering a foundation for more effective and individualized therapeutic approaches, our study has the potential to improve the care and management of AD patients across subtypes.

## CONFLICT OF INTEREST STATEMENT

The authors declare no competing interests. Author disclosures are available in the supporting information.

## CONSENT STATEMENT

The study adhered to the Declaration of Helsinki and was approved by the IRBs of all participating centers. Written informed consent was obtained from all participants or their authorized representatives.

## CODE AVAILABILITY STATEMENT

All software used in this study is publicly available. The code used in this study can be accessed at https://github.com/wzhang96/AD_Subtypes/tree/master.

## Supporting information



Supporting Information

Supporting Information

Supporting Information

## Data Availability

The data used in this study were obtained from the ADNI database (adni.loni.usc.edu) and the PPMI database (www.ppmi‐info.org). Access to both datasets is subject to data use agreements and requires registration and approval. Researchers can apply for ADNI data through the ADNI website and for PPMI data through the PPMI website. Detailed information on data collection procedures and available datasets can be found on the respective portals. All other data are available from the corresponding authors of the original publications upon reasonable request.
