# Supplementary material for: Proteomics reveals three molecular subtypes of Alzheimer's disease with distinct progression patterns
Source: Alzheimers Dement. 2026 Feb 6;22(2):e71106. doi: 10.1002/alz.71106 (PMC12880608; doi:10.1002/alz.71106)
Supplement: Supplementary file 1 — Supporting Information [file ALZ-22-e71106-s003.docx]

**Proteomic Profiling Uncovers Three Alzheimer's Subtypes with Distinct Molecular Signatures and Progression Trajectories**

**Supplementary Figures**

**Supplementary Figure 1.** Flowchart of independent validation using random forest classification.

**Supplementary Figure 2.** Comparison of CSF Aβ, total tau, and p-tau levels across three subtypes stratified by APOE ε4 carrier status.

**Supplementary Figure 3.** Comparison of cognitive profiles across the three subtypes after adjusting for CSF Aβ.

**Supplementary Figure 4.** CSF biomarkers and brain atrophy features of the three subtypes after adjusting for CSF Aβ.

**Supplementary Figure 5.** Comparison of CSF Aβ, total tau, and p-tau levels across three subtypes in MCI participants.


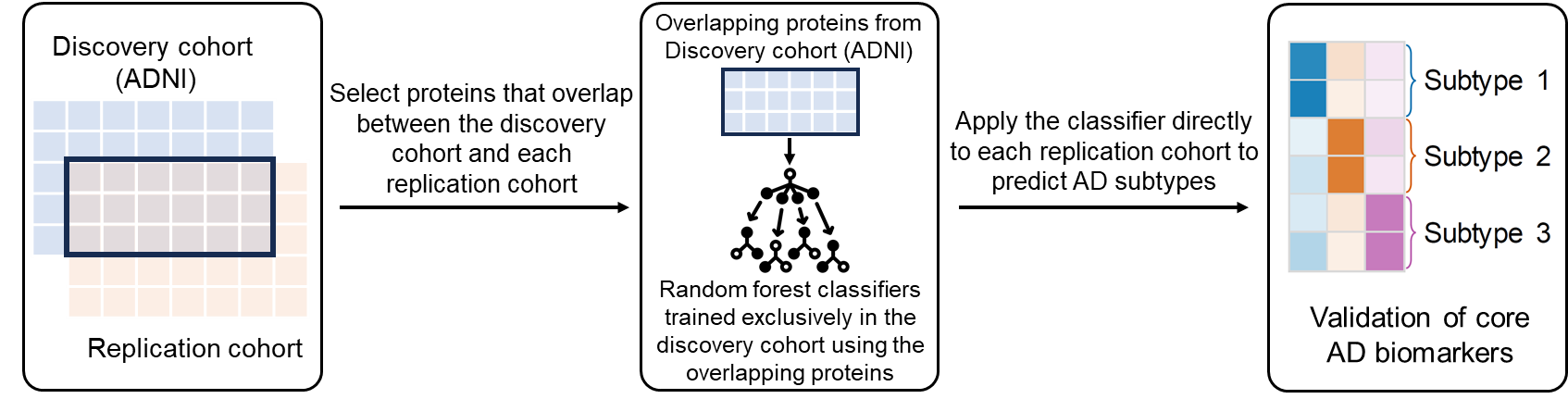


**Supplementary Fig. 1| Flowchart of independent validation using random forest classification.** Random forest classifiers were trained exclusively in the discovery cohort (ADNI) using proteins that overlapped with each replication dataset. The trained models were then directly applied to the replication cohorts to predict subtype membership, without incorporating any replication data during model training.

**Supplementary Figures**


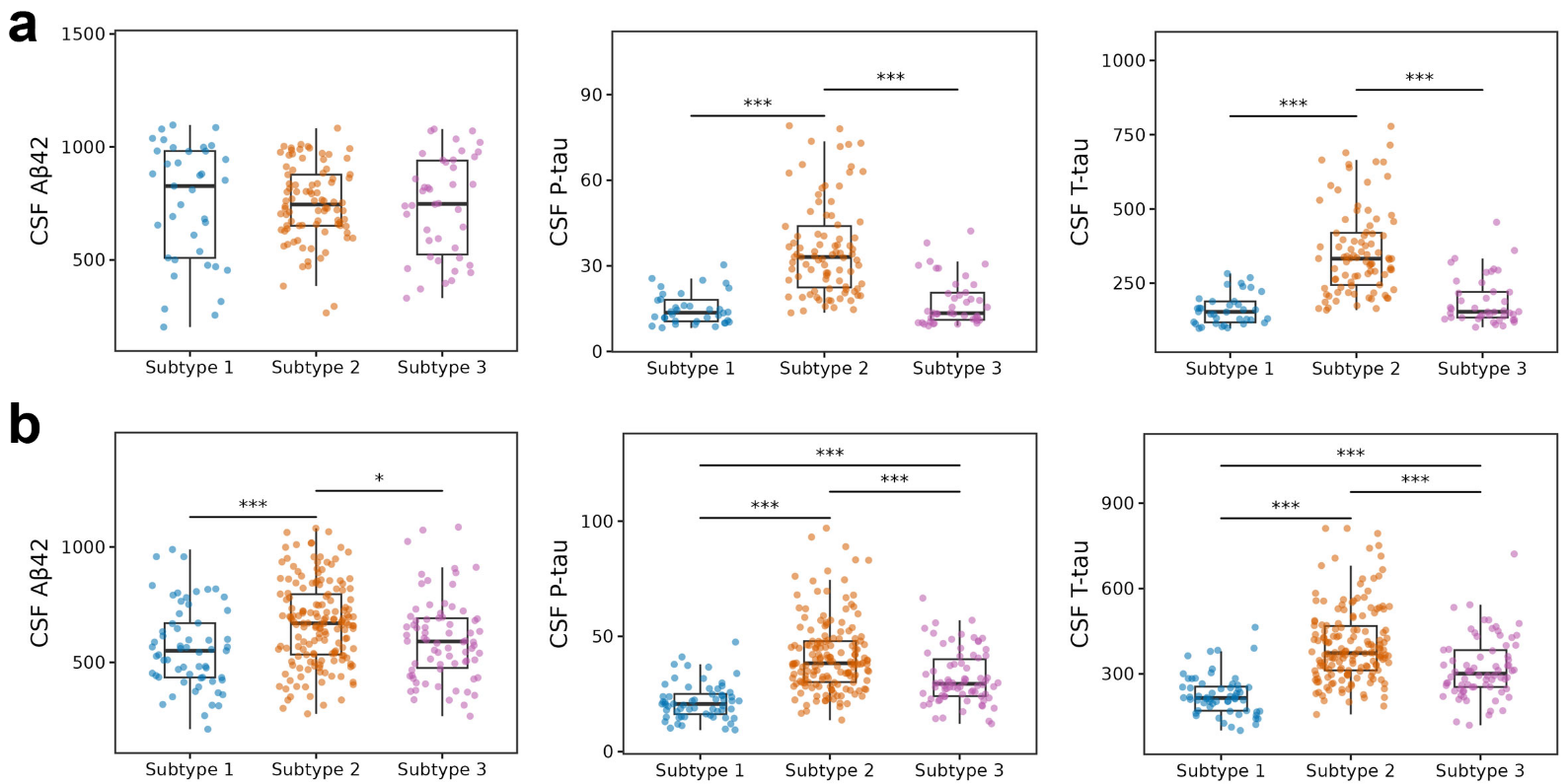


**Supplementary Fig. 2| Comparison of CSF Aβ, total tau, and p-tau levels across three subtypes stratified by APOE ε4 carrier status. (a)** In APOE ε4 non-carriers, subtype 2 exhibited significantly higher levels of p-tau and t-tau compared to subtype 1 and subtype 3. **(b)** In APOE ε4 carriers, CSF Aβ, p-tau, and t-tau levels were highest in subtype 2. Aβ levels did not differ significantly between subtype 1 and subtype 3, whereas both p-tau and t-tau levels were higher in subtype 3 than in subtype 1.


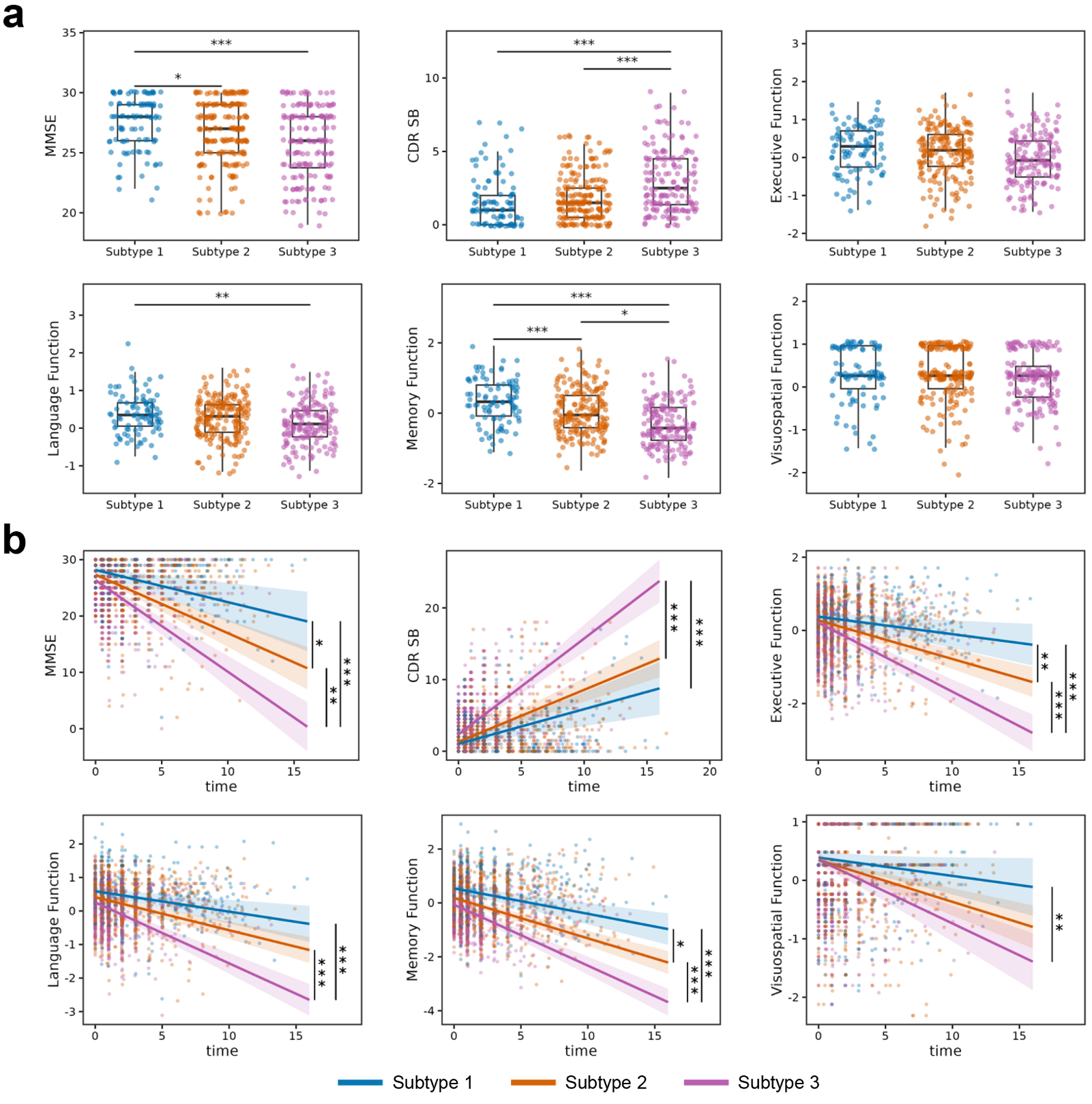


**Supplementary Fig. 3| Comparison of cognitive profiles across the three subtypes after adjusting for CSF Aβ. (a)** Baseline cognitive functions among the three subtypes. Significant differences were observed in MMSE, CDR-SB, language function, and memory function at baseline among the subtypes, while executive function, visuospatial function did not show significant differences. **(b)** Cognitive decline during the follow-up period differed significantly among the subtypes across multiple measures, including MMSE, CDR-SB, executive function, language function, memory function, and visuospatial function.


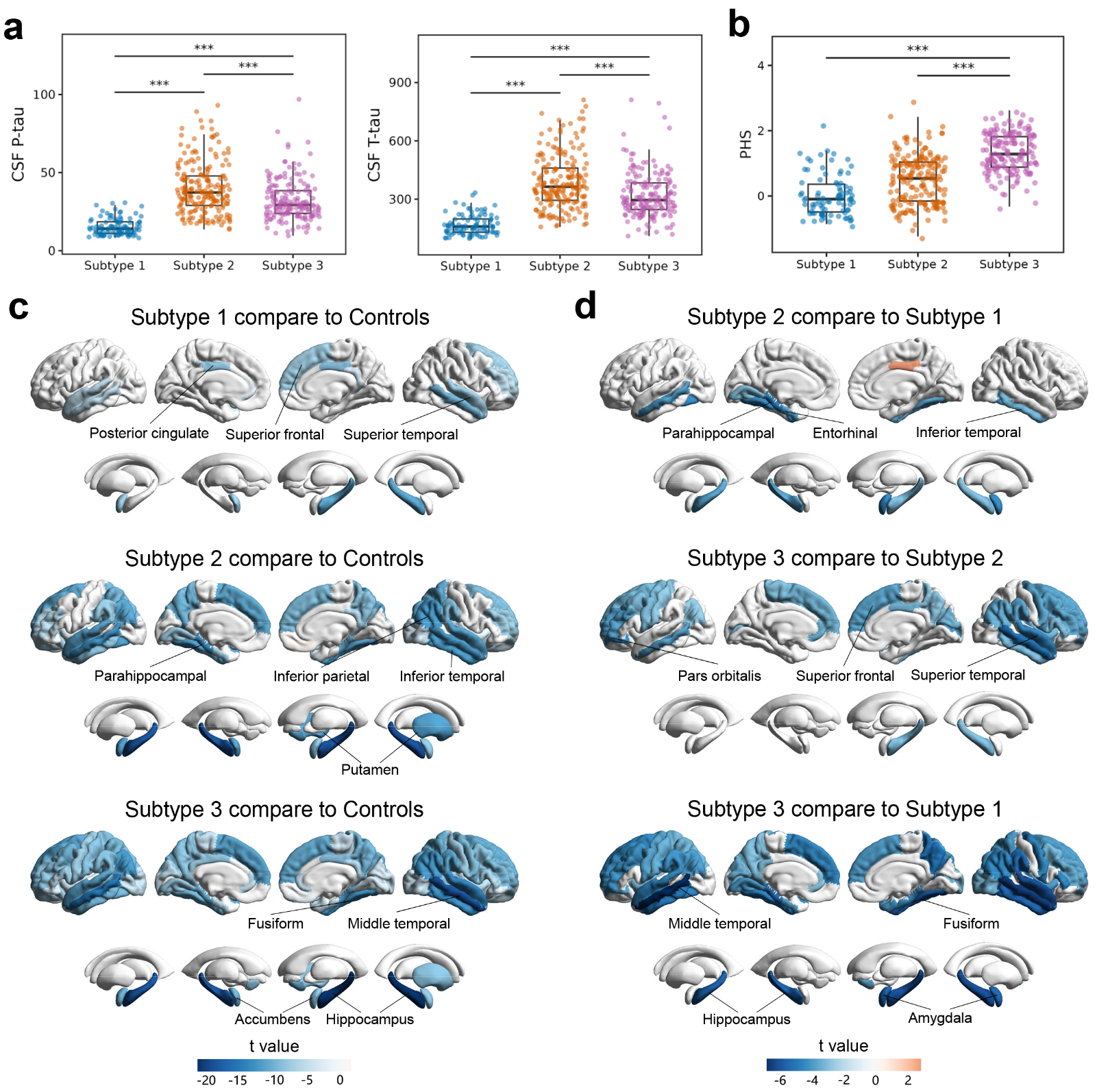


**Supplementary Fig. 4| CSF biomarkers and brain atrophy features of the three subtypes after adjusting for CSF Aβ. (a)** Levels of CSF p-tau, and t-tau among the three subtypes. CSF p-tau and t-tau levels were highest in subtype 2, with subtype 3 showing intermediate levels. For p-tau, subtype 2 had significantly higher levels than subtype 1 (*p* = 2.48 × 10⁻³²), and also higher than subtype 3 (*p* = 3.75 × 10⁻⁹). Subtype 3 showed significantly higher levels than subtype 1 as well (*p* = 3.75 × 10⁻⁹). For t-tau, the same pattern was observed: subtype 2 had significantly higher levels than subtype 1 (*p* = 7.23 × 10⁻³⁴) and subtype 3 (*p* = 6.76 × 10⁻⁹). Subtype 3 also had significantly higher t-tau levels than subtype 1 (*p* = 4.08 × 10⁻¹⁰). **(b)** Differences in Polygenic Hazard Scores (PHS) among the three subtypes. Subtype 3 showed the highest PHS. **(c)** Comparison of longitudinal brain atrophy rates between the three subtypes and the control group. Compared to the control group, the three subtypes exhibited different atrophy rates in brain regions, with subtype 3 showing the most severe longitudinal brain atrophy and subtype 1 showing the mildest. **(d)** Comparison of longitudinal brain atrophy rates among the three subtypes. Among the three subtypes, subtype 3 exhibited the fastest brain atrophy rates, followed by subtype 2, while subtype 1 showed the slowest progression.


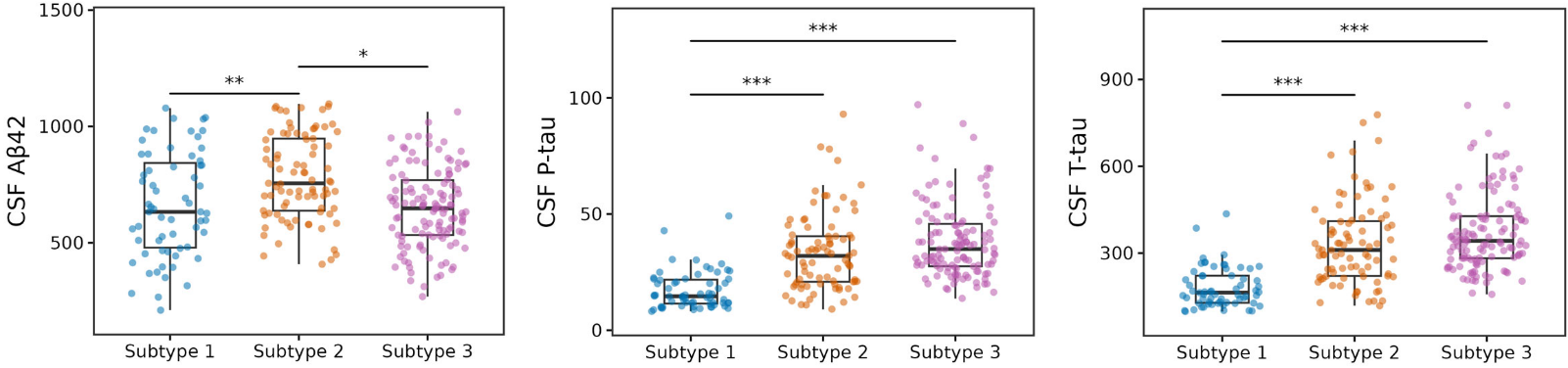


**Supplementary Fig. 5| Comparison of CSF Aβ, total tau, and p-tau levels across three subtypes in MCI participants.** CSF levels of Aβ were higher in subtype 2 than in subtype 1 and subtype 3. Subtype 2 exhibited substantially higher levels of both p-tau and t-tau compared to subtype 1. Although p-tau and t-tau levels in subtype 2 were also higher than those in subtype 3, these differences did not reach statistical significance.
